# Evaluation of farmers’ diagnostic performance for detection of diarrhoea in nursery pigs using digital pictures of faecal pools

**DOI:** 10.1186/1751-0147-55-72

**Published:** 2013-10-18

**Authors:** Ken S Pedersen, Anne M Strunz

**Affiliations:** 1 HERD – Centre for Herd-oriented Education, Research and Development, Department of Large Animal Sciences, Faculty of Health and Medical Sciences, University of Copenhagen, Groennegaardsvej 2, Frederiksberg, DK-1870 Copenhagen, Denmark; 2 Ø-VET Næstved A/S, Køberupvej 33, DK-4700 Næstved, Denmark

**Keywords:** Clinical signs, Diagnostic performance, Farmers, Faecal consistency, Diarrhoea, Pigs

## Abstract

**Background:**

Overconsumption of antibiotics in the pig industry is of concern in relation to antimicrobial resistance. False positive disease diagnosis may result in the treatment of healthy animals. In Denmark, diarrhoea is the most common cause of antibiotic treatment in pigs. Farm personnel are not professional clinicians, which could result in inappropriate antibiotic treatments of diarrhoea.

The primary objectives of this pilot study using digital pictures of faecal pools was to evaluate farmers’ diagnostic performance in the assessment of faecal consistency in nursery pigs and to investigate the effect of different co-variables, including practical experience. A secondary objective was to compare the diagnostic performance of farmers with that of veterinarians.

At a pig congress, observers (farm personnel and veterinarians) working professionally with pigs participated in a faecal consistency test consisting of 16 pictures of faecal pools (eight diarrhoeic and eight non-diarrhoeic). The faecal pools had previously been collected and subjected to faecal dry matter determination. The true status of the faecal pools was determined by the faecal dry matter content (diarrhoea: faecal dry matter ≤ 18%). The true status was used to evaluate the farmers’ and veterinarians’ diagnostic performance.

**Results:**

A total of 119 farmers and 18 veterinarians were included in the statistical analysis. For the farmers, the mean proportion of faecal pools assessed as diarrhoeic was 0.48, the mean proportion of correctly classified faecal pools was 0.84, the mean diagnostic sensitivity was 0.83 and the mean diagnostic specificity was 0.86. Farmers with less than four years of practical experience detected clinical diarrhoea more accurately than farmers with more than four years of practical experience (p < 0.05). No significantly differences between farmers and veterinarians was observed (p > 0.20).

**Conclusions:**

The results, using digital pictures of faecal pools, suggest that farmers and veterinarians have similar diagnostic performance in relation to diarrhoea. False positive classification of non-diarrhoeic pigs appears to be a larger problem than false negative classification of diarrhoeic pigs under Danish conditions. If these results can be confirmed under practical conditions, training in, and validation of, clinical diagnoses may be an important factor in reducing antibiotic consumption in the pig industry.

## Background

Overconsumption of antibiotics in the pig industry is of concern due to antimicrobial resistance in both animals and humans [1]. A potential cause of excessive use of antibiotics is incorrect clinical diagnosis of diseases, resulting in false positive disease diagnosis and treatment of healthy animals.

In Denmark, approximately 35% of the antibiotic consumption in pigs is used for the treatment of intestinal diseases in nursery pigs [2]. Diarrhoea is an important clinical sign of intestinal diseases.

In human medicine, diarrhoea may be defined in terms of stool frequency, consistency, volume, or weight [3]. In veterinary medicine, diarrhoea has been defined as an increased frequency of defecation accompanied by faeces containing an increased concentration of water and decreased dry matter content [4]. In both diarrhoea definitions, faecal consistency is a key characteristic, and, the evaluation of faecal consistency is important during a clinical examination of pigs to identify diarrhoea both at individual and herd level. Additional faecal abnormalities include changes in colour, smell and/or admixture of blood, mucus and/or necrotic material [4]. Changes in relation to these additional characteristics are not included in the diarrhoea definitions [3,4].

Clinical assessment of diarrhoea in pigs is subject to inter-observer variation [5]. In the assessment of faecal consistency, a low level of inter-observer variation has been demonstrated in human medicine [6], while both intra- and inter-observer variations have been demonstrated in relation to faecal consistency in growing pigs [7].

In Denmark, farm personnel perform the clinical assessment and decide whether or not to initiate antibiotic treatment of animals. Farm personnel are not professional clinicians and have not undergone the same level of clinical diagnostic training. Poor diagnostic ability of farm personnel when assessing faecal consistency could potentially result in false clinical diagnoses of diarrhoea and biased antibiotic treatments of diarrhoea. We are unaware of any published studies on farmers’ diagnostic performance in the assessment of diarrhoea defined by an abnormal faecal consistency.

Therefore, the primary objective of this pilot study using digital pictures of faecal pools was to evaluate farmers’ diagnostic performance in the assessment of faecal consistency in nursery pigs and to investigate the effect of different co-variables, including practical experience. A secondary objective was to compare the diagnostic performance of farmers with that of veterinarians.

## Methods

### Design and sample size considerations

Observers (farm personnel - “farmers” - and veterinarians) working professionally with pigs participated in a faecal consistency test. The test was performed using digital pictures of faeces as previously described for inter-observer agreement studies in human medicine [8].

The diagnostic ability of each participant was evaluated by calculating the assessed diarrhoea prevalence, the proportion of correctly classified faecal pools and diagnostic sensitivity and specificity. No formal sample size calculations were performed in relation to the number of pictures in the faecal consistency test. A time slot of 20 minutes was provided at the congress. Therefore, 16 pictures were included in the faecal consistency test.

### Selection of observers

The observers were selected by convenience to represent Danish farm personnel and pig veterinarians. In order to include a larger number of observers, it was decided to perform the consistency test during a national pig congress. A national pig congress held in the eastern part of Denmark in January 2011 was selected. The participants included pig farm personnel and pig veterinarians primarily from the eastern part of Denmark. Approximately 125 persons working on pig farms and 20 pig veterinarians were expected to participate in the congress. All participants attending the congress were included in the study.

### Pictures and collection of faecal pools

A Danish herd with a history of *Lawsonia intracellularis*-associated diarrhoea was selected. The herd contained 650 sows and 2500 nursery pigs and used dry feeding *ad libitum* based on soybean, barley and wheat. In the nursery, each pen contained approximately 40 pigs and had partially slatted floors.

All faecal pools from nursery pigs included in the faecal consistency test were photographed and collected on the farm between two and ten weeks post weaning (approximately six to 14 weeks old). The faecal pools were selected from among freshly deposited faeces on the floor of the pens. The faecal pools were selected by non-random purposive sampling [9] to include a range of different faecal consistencies in the test. A previously described [7] faecal consistency scale with 4 descriptive categories and explanations in text and pictures was used during the selection process in order to assist in the selection of different consistencies. The four consistency categories were: score 1 = firm and shaped, score 2 = soft and shaped, score 3 = loose, and score 4 = watery. Scores 1 and 2 were considered normal, while scores 3 and 4 were considered as diarrhoea.

Following the selection of a faecal pool, a digital picture was taken using a Canon EOS 400D 10.5 megapixel digital camera with a Canon EF-S 18-55 mm standard zoom lens and automatic flash (Canon Zhuhai Inc., Zhuhai, China). All pictures were taken at a distance of 20–30 cm from each faecal pool.

Immediately after the individual pictures had been taken, a faecal sample of minimum 5 grams of faeces was collected from each faecal pool. The faecal samples were kept in plastic faecal containers (height 7 cm, diameter 4.5 cm, LVKVilofarm Online shop, trade name: Salmonella Manure box red, item number 1152) closed with a lid and marked with an identification number. A total of 20 faecal pools were photographed and collected.

One observer (corresponding author) performed the selection and collection of the faecal samples.

### Processing of faecal samples and selection of pictures

The faecal samples were transported by car to the National Veterinary Institute, Technical University of Denmark on the same day as the herd visit. The faecal samples were stored overnight at 4°C and further processed the following day. Each of the obtained faecal samples was subjected to faecal dry matter determination using the microwave procedure as previously described [10]. One person (corresponding author) performed all faecal dry matter determinations.

The true diarrhoea status of the selected and photographed faecal pools was determined using the faecal dry matter content (diarrhoea: faecal dry matter ≤ 18%) [10]. Following the faecal dry matter determination and quality assessment of the pictures a total of 16 pictures were selected for the faecal consistency test. The 16 pictures were selected to include a range of different faecal consistencies in the test. A total of eight pictures showed diarrhoeic faecal pools (see Additional file 1), and eight pictures showed normal faecal pools (see Additional file 2), providing a true diarrhoea prevalence of 50% in the faecal consistency test.

### Performing the faecal consistency test

The faecal consistency test was performed once in an auditorium where all participants at the congress were present. Prior to the faecal consistency test, all participants were instructed in the plenary session to rate the faecal pool on each picture as diarrhoea or not on a registration form. During the faecal consistency test the pictures were in random order. Each picture was marked with a number and shown for approximately one minute on a big screen (4 × 4 m) using Microsoft PowerPoint 2010. The participants were not allowed to discuss the pictures. The participants were requested to provide additional information on the registration form. This information included which part of the production phase they were working with on a daily basis and what practical experience they had had with pig production. The practical experience data (the total number of years a person had been working with pigs) were obtained as a categorical variable dichotomised at 4 years. The 4 years of experience was selected because it is equivalent to the duration of a farmer’s education obtained at an agricultural college in Denmark.

### Statistical analysis

The true diarrhoea status of the faecal pools (defined by the faecal dry matter content) was used to calculate the diagnostic sensitivity, specificity and proportion of correctly classified faecal pools for each participant. Furthermore, the proportion of pools assessed as diarrhoeic was calculated for each participant. These different diagnostic measures were compared between farmers and veterinarians. The effect of the co-variables’ practical experience and production phase (1: currently working with sows only on a daily basis, 2: working with finishers or a combination of finishers and sows, 3: working with nursery pigs only or nursery pigs in combination with sows and/or finishers) was evaluated for the farmers. The statistical evaluation was performed using generalised linear mixed models with participant and picture included as crossed random effects. The xtmelogit command in Stata IC/11 was used.

## Results

A total of 141 persons at the congress participated in the test. The participants included 122 persons working on pig farms, 18 pig veterinarians and one animal scientist. The animal scientist was excluded from further analysis along with two farmers with missing values (missing number of years working with pigs) resulting in a total of 119 farmers and 18 veterinarians for the statistical analysis.

For the farmers, 17% (n = 20) had been working with pigs for less than four years, while 83% (n = 99) had been working with pigs for more than four years. A total of 18% (n = 22) of the farmers were currently working with sows only on a daily basis, 18% (n = 21) worked with finishers or a combination of finishers and sows, while 64% (n = 76) worked with nursery pigs only or nursery pigs in combination with sows and/or finishers.

The diagnostic measures for both farmers and veterinarians are displayed in Table 1. The statistical analyses demonstrated that none of the diagnostic measures were significantly different between farmers and veterinarians (p > 0.20), (Table 1).

**Table 1:** Diagnostic performance of farmers and veterinarians

		**Mean**	**SD**
Proportion of faecal pools assessed as diarrhoeic	Farmers	0.48	0.11
	Veterinarians	0.48	0.11
Proportion of correctly classified faecal pools*	Farmers	0.84	0.09
	Veterinarians	0.87	0.07
Diagnostic sensitivity for detection of diarrhoeic faecal pools*	Farmers	0.83	0.16
	Veterinarians	0.85	0.14
Diagnostic specificity for detection of diarrhoeic faecal pools*	Farmers	0.86	0.13
	Veterinarians	0.89	0.11

Mean (SD = standard deviation) diagnostic performance of farmers (n = 119) and veterinarians (n = 18) in assessment of diarrhoea in growing pigs using 16 digital pictures of faecal pools.

*For each observer the diagnostic sensitivity was calculated as the number of digital pictures assessed as diarrhoeic divided by the true number of digital pictures displaying diarrhoeic faecal pools. The diagnostic specificity was calculated as the number of digital pictures assessed as normal divided by the true number of digital pictures displaying normal faecal pools. Faecal dry matter ≤18% was reference standard for classification of individual faecal pools.

For the participating farmers the number of years working with pigs was the only co-variable observed to be significantly associated with diagnostic performance. The four diagnostic measures and associated p-values stratified by the number of years working with pigs are displayed in Table 2.

**Table 2:** Association between number of years working with pigs and diagnostic performance

**Diagnostic measure**	**Working with pigs**	**Mean (SD)**	**p-value**^**#**^
Proportion of faecal pools assessed as diarrhoeic	< 4 years	0.53 (0.08)	0.056
	≥ 4 years	0.48 (0.12)	
Proportion of correctly classified faecal pools*	< 4 years	0.88 (0.06)	0.017
	≥ 4 years	0.84 (0.09)	
Diagnostic sensitivity for detection of diarrhoeic faecal pools*	< 4 years	0.91(0.11)	0.004
	≥ 4 years	0.81(0.16)	
Diagnostic specificity for detection of diarrhoeic faecal pools*	< 4 years	0.86 (0.10)	0.87
	≥ 4 years	0.86 (0.13)	

^#^Result from generalized linear mixed models.

*For each observer the diagnostic sensitivity was calculated as the number of digital pictures assessed as diarrhoeic divided by the true number of digital pictures displaying diarrhoeic faecal pools. The diagnostic specificity was calculated as the number of digital pictures assessed as normal divided by the true number of digital pictures displaying normal faecal pools. Faecal dry matter ≤18% was reference standard for classification of individual faecal pools.

Association between the total number of years a farmer had been working with pigs and the diagnostic performance for farmers (n = 119) in assessment of diarrhoea in growing pigs using 16 digital pictures of faecal pools.

## Discussion

Variation in clinical assessment between farmers is well recognised in veterinary practice and has also been reported in the scientific literature [11]. Differences between farmers and scientists have previously been reported for the assessment of locomotion scores in sows [12], while non-veterinarians showed good diagnostic performance in the assessment of injuries in horses [13].

However, a small number of publications indicate that farmers’ diagnostic performance and intra- and inter-observer agreement appear not to have been quantitatively evaluated to the same extent as the differences between veterinarians.

The results of our study demonstrated that the farmers’ ability to classify correctly pictures of faecal pools is not different to that of veterinarians. This suggests that the farmers’ diagnostic ability to detect clinical diarrhoea may be similar to that of veterinarians. This further suggests that average farmers and veterinarians will apply antibiotics for treatment of diarrhoea to the same pigs. However, the observed imperfect diagnostic performance of both farmers and veterinarians suggests that the treatment of healthy animals and the non-treatment of sick animals will occur in practice. The occurrence of these in-correct treatments will depend on the true diarrhoea prevalence and the medication strategy. In relation to individual treatments (e.g. treatment by injection of single animals), it is the clinical examination and classification of the individual pigs that will determine whether the pigs will be subjected to antibiotic treatment. In a population with a high prevalence of diarrhoea (e.g. 80%), the observed diagnostic sensitivity for both farmers and veterinarians will result in a large number of diarrhoeic pigs being falsely classified as diarrhoea-negative (low negative predictive value). However in a population with a medium to low prevalence of diarrhoea (e.g. <30%), the observed diagnostic specificity for both farmers and veterinarians will result in a large number of non-diarrhoeic pigs being falsely classified as diarrhoea-positive (low positive predictive value). There are currently no published data on diarrhoea prevalence in Danish nursery pigs. However, low diarrhoea prevalence has previously been reported in Danish finishers [14]. This implies that under Danish conditions false positive classification of pigs as diarrhoeic is a larger problem than false negative classification of pigs as non-diarrhoeic. This could potentially result in overconsumption of antibiotics used for individual treatments.

In relation to the application of batch medication, it is likely the prevalence of diarrhoea in the group of pigs that will be part of the process to determine whether the pigs will be subjected to antibiotic treatment. The observed diagnostic sensitivity and specificity will also result in biased estimation of the diarrhoea prevalence in a group of pigs. In a population with a high true diarrhoea prevalence (e.g. 80%), the apparent diarrhoea prevalence will be marginally underestimated, equivalent to the apparent diarrhoea prevalence of 0.48 observed in the faecal consistency test (true diarrhoea prevalence = 0.50). In contrast, the prevalence will be overestimated in populations with a medium to low true diarrhoea prevalence (e.g. <30%). This implies an overuse of batch medication for diarrhoea under Danish conditions similar to the application of individual treatments.

The variation in diagnostic performance between farmers demonstrated that some farmers have a poor diagnostic ability. Studies of factors explaining such differences between farmers could form a basis for identifying or predicting farmers who need increased supervision or clinical training. In the current study, the number of years working with pigs was the only co-variable associated with the farmers’ diagnostic performance, and the effect was low. The less experienced farmers had a diagnostic performance comparable to that of veterinarians, while the more experienced farmers actually had a poorer diagnostic ability and were more reluctant to assess faecal pools as diarrhoeic. A similar effect of increasing experience has previously been reported to be associated with diagnostic performance of lameness in cattle [15]. A possible explanation could be that less experienced personnel have been trained more recently by veterinarians in making clinical diagnoses.

A potential factor not investigated in the current study is the effect of farm prevalence of clinical signs. Farmers may adapt to the level of a disease. In that way, farmers from high diarrhoea prevalence farms may be more reluctant to classify a pig as being diarrhoeic. However, this aspect needs to be confirmed.

Another aspect that needs further consideration is that it has previously been demonstrated that decisions relating to the treatment of animals are dependent on factors other than the simple assessment of clinical signs [16].

On-farm examination of faecal pools would be preferable to the pictures used in this study. However, faecal pools are difficult to preserve over longer periods of time, and therefore the inclusion of larger numbers of observers would be impossible. We used digital pictures of faecal pools in order to include a larger number of observers so the study would be more representative. The digital pictures were used as a proxy for real faecal pools similar to the evaluation of inter-observer agreement in relation to the assessment of faeces in human medicine [8]. The use of digital pictures has been evaluated most intensively in dermatology. In dermatology, the examination of digital pictures has a high level of agreement compared with the physical examination of dermatological lesions [17]. Furthermore, the application of digital pictures has been reported to reduce bias more than physical examination in dermatological studies [18].

All faecal samples were collected from the same herd. Theoretically, this could have increased or decreased the observed agreements if faecal samples from the selected herd had a unique consistency or appearance. This bias is highly unlikely, since inter-observer agreement and observer accuracy for the assessment of faecal consistency in nursery pigs have been demonstrated to be consistent between herds [9,19].

## Conclusions

The results of the pilot study suggest that farmers and veterinarians have similar diagnostic performance in relation to clinical diarrhoea. A large variation between farmers was observed, with less experienced farmers having a better diagnostic performance.

The results of the pilot study suggest that false positive classification of non-diarrhoeic pigs is a larger problem than false negative classification of diarrhoeic pigs under Danish conditions. The findings suggest that training in, and validation of, clinical diagnoses is important in order to reduce the use of antibiotics in the pig industry.

## Competing interest

The authors declare that they have no competing interests.

## Authors’ contributions

Both authors conceived and designed the study. KSP performed all of the work relating to the faecal samples. Both authors were involved in performing the faecal tests. KSP conducted the statistical analyses. Both authors participated in drafting the manuscript. Both authors have read and approved the final manuscript.

## Supplementary Material

Additional file 1 Eight digital pictures of porcine diarrhoeic faecal pools used for evaluation of farmers’ diagnostic performance for detection of diarrhoea in nursery pigs. Click here for file

Additional file 2 Eight digital pictures of porcine normal faecal pools used for evaluation of farmers’ diagnostic performance for detection of diarrhoea in nursery pigs. Click here for file
