# Supplementary material for: Evaluation of farmers’ diagnostic performance for detection of diarrhoea in nursery pigs using digital pictures of faecal pools
Source: Acta Vet Scand. 2013 Oct 18;55(1):72. doi: 10.1186/1751-0147-55-72 (PMC3819656; doi:10.1186/1751-0147-55-72)

Additional file 1. Eight digital pictures of porcine diarrhoeic faecal pools used for evaluation of farmers' diagnostic performance for detection of diarrhoea in nursery

Picture 2

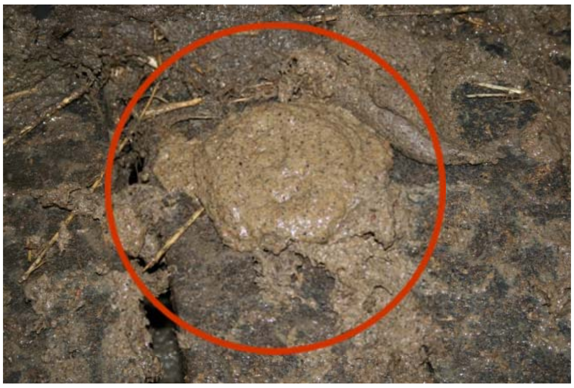

Picture 4

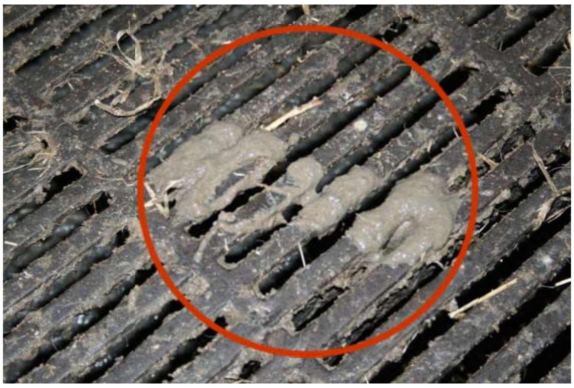

Picture 7

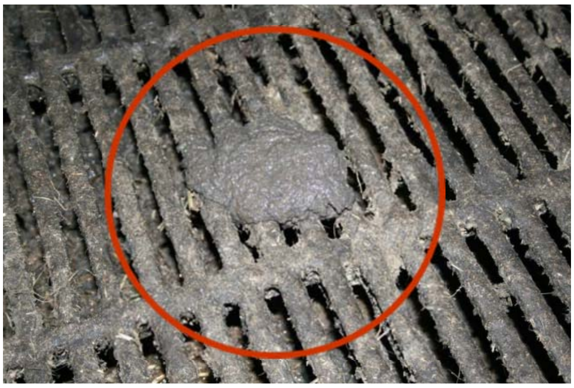

Picture 10

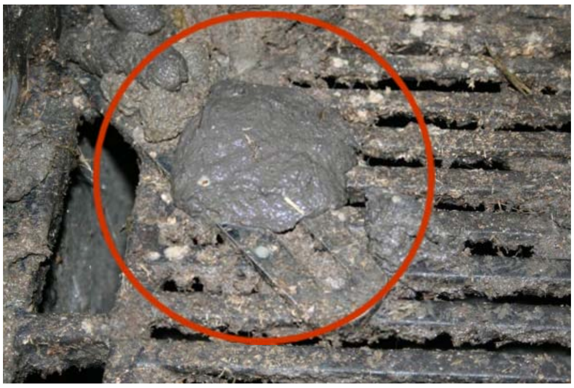

Picture 11

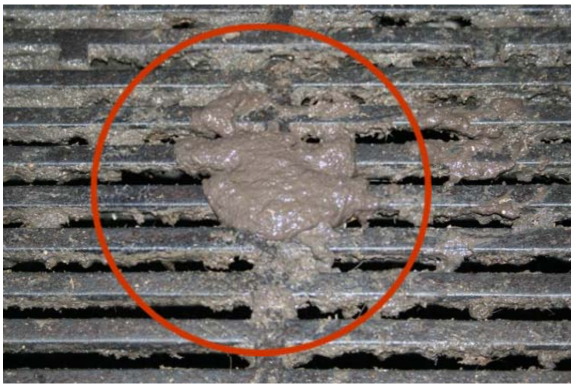

Picture 13

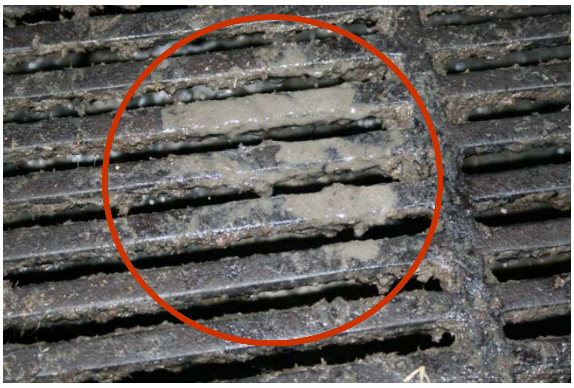

Picture 15

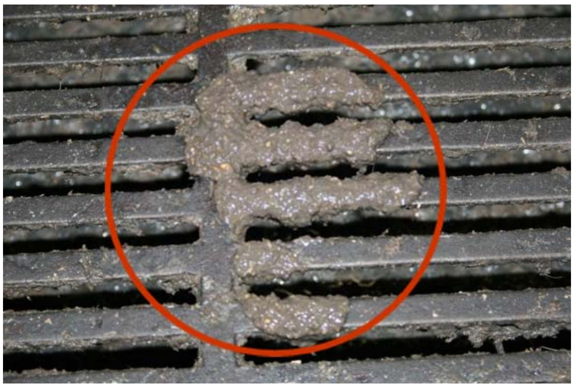

Picture 16

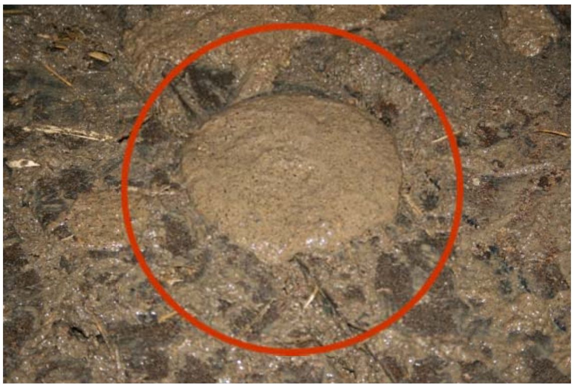

Supplement: Additional file 1 — Eight digital pictures of porcine diarrhoeic faecal pools used for evaluation of farmers’ diagnostic performance for detection of diarrhoea in nursery pigs. [file 1751-0147-55-72-S1.pdf]
