# Supplementary material for: Evaluation of farmers’ diagnostic performance for detection of diarrhoea in nursery pigs using digital pictures of faecal pools
Source: Acta Vet Scand. 2013 Oct 18;55(1):72. doi: 10.1186/1751-0147-55-72 (PMC3819656; doi:10.1186/1751-0147-55-72)

Additional file 2. Eight digital pictures of porcine normal faecal pools used for evaluation of farmers' diagnostic performance for detection of diarrhoea in nursery

Picture 1

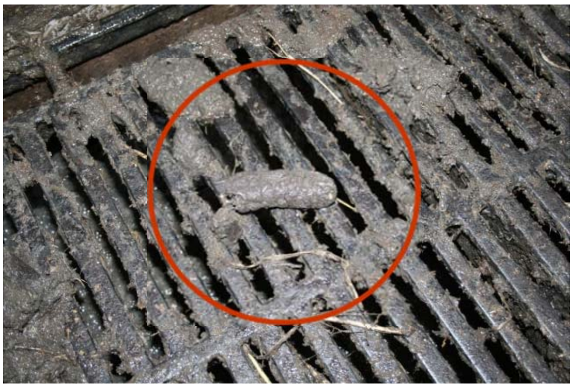

Picture 3

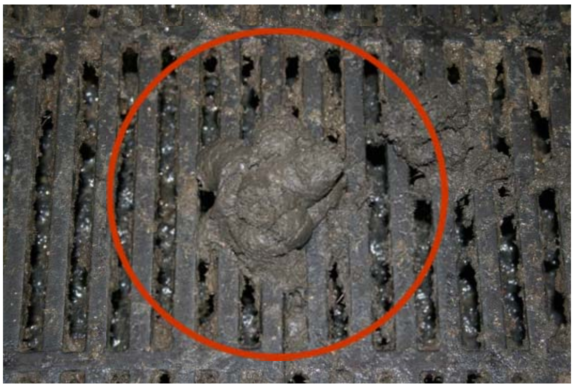

Picture 5

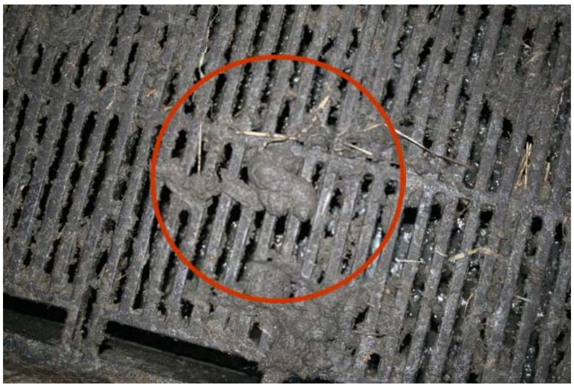

Picture 6

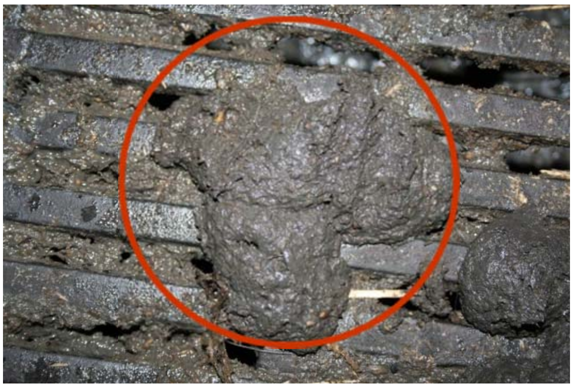

Picture 8

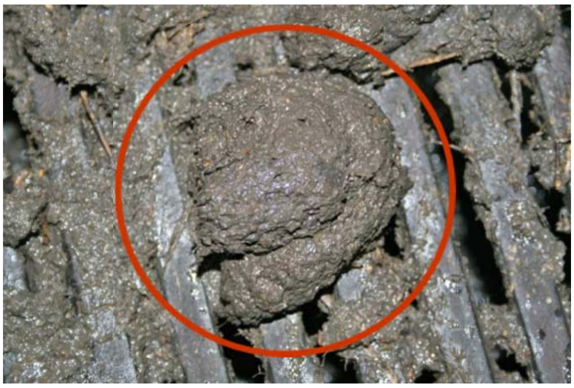

Picture 9

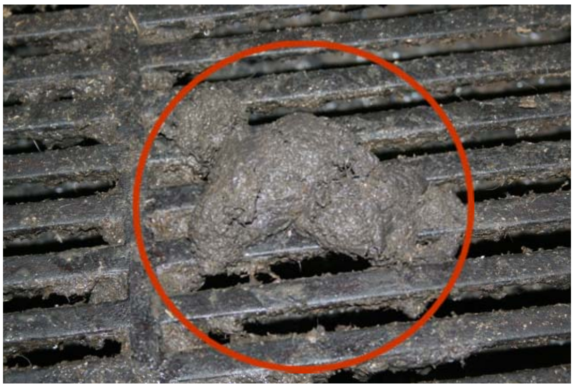

Picture 12

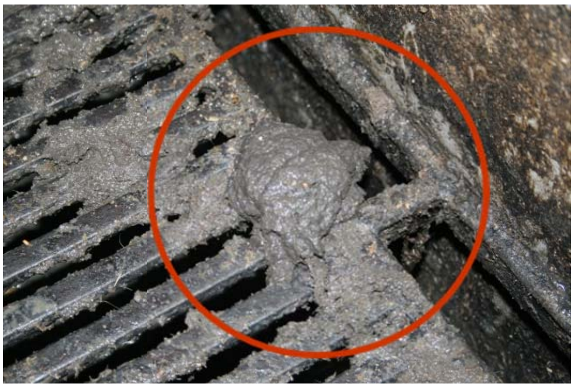

Picture 14

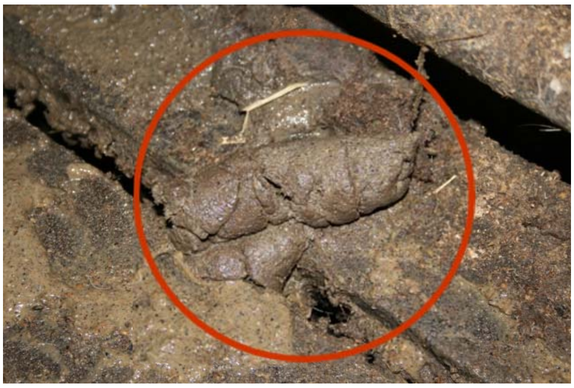

Supplement: Additional file 2 — Eight digital pictures of porcine normal faecal pools used for evaluation of farmers’ diagnostic performance for detection of diarrhoea in nursery pigs. [file 1751-0147-55-72-S2.pdf]
